# Antibody-Dependent Cell-Mediated Cytotoxicity Epitopes on the Hemagglutinin Head Region of Pandemic H1N1 Influenza Virus Play Detrimental Roles in H1N1-Infected Mice

**DOI:** 10.3389/fimmu.2017.00317

**Published:** 2017-03-21

**Authors:** Zi-Wei Ye, Shuofeng Yuan, Kwok-Man Poon, Lei Wen, Dong Yang, Zehua Sun, Cun Li, Meng Hu, Huiping Shuai, Jie Zhou, Mei-Yun Zhang, Bo-Jian Zheng, Hin Chu, Kwok-Yung Yuen

**Affiliations:** ^1^Department of Microbiology, The University of Hong Kong, Hong Kong; ^2^State Key Laboratory of Emerging Infectious Diseases, The University of Hong Kong, Hong Kong; ^3^Research Centre of Infection and Immunology, The University of Hong Kong, Hong Kong; ^4^Carol Yu Centre for Infection, The University of Hong Kong, Hong Kong

**Keywords:** antibody-dependent cell-mediated cytotoxicity, H1N1 influenza virus, hemagglutinin, lung damage, mice

## Abstract

Engaging the antibody-dependent cell-mediated cytotoxicity (ADCC) for killing of virus-infected cells and secretion of antiviral cytokines and chemokines was incorporated as one of the important features in the design of universal influenza vaccines. However, investigation of the ADCC epitopes on the highly immunogenic influenza hemagglutinin (HA) head region has been rarely reported. In this study, we determined the ADCC and antiviral activities of two putative ADCC epitopes, designated E1 and E2, on the HA head of a pandemic H1N1 influenza virus *in vitro* and in a lethal mouse model. Our data demonstrated that sera from the E1-vaccinated mice could induce high ADCC activities. Importantly, the induction of ADCC response modestly decreased viral load in the lungs of H1N1-infected mice. However, the elevated ADCC significantly increased mouse alveolar damage and mortality than that of the PBS-vaccinated group (*P* < 0.0001). The phenotype was potentially due to an exaggerated inflammatory cell infiltration triggered by ADCC, as an upregulated release of cytotoxic granules (perforin) was observed in the lung tissue of E1-vaccinated mice after H1N1 influenza virus challenge. Overall, our data suggested that ADCC elicited by certain domains of HA head region might have a detrimental rather than protective effect during influenza virus infection. Thus, future design of universal influenza vaccine shall strike a balance between the induction of protective immunity and potential side effects of ADCC.

## Introduction

Influenza viruses, as one of the major zoonotic pathogens, have continuously caused global health concerns due to their high propensity for unpredictable genetic mutation in major surface antigens, hemagglutinin (HA), and neuramindase. Antivirals and vaccines are vital in combating the threat of influenza epidemics and pandemics. However, the increasing usage of licensed antivirals has resulted in the global emergence of amantadine- and/or oseltamivir-resistant strains of influenza virus. Typical examples include the worldwide spread of adamantine resistant A(H3N2) viruses since 2003, oseltamivir-resistant seasonal A(H1N1) viruses since 2007, adamantane-resistant pandemic A(H1N1) viruses in 2009, and peramivir-resistant A(H7N9) viruses in 2013 (1). On the other hand, seasonal influenza vaccines have to be updated annually due to the continuous antigenic drift and shift (2). Otherwise, the mismatch between vaccinated formulations and that of natural selection would considerably limit the effectiveness of influenza vaccines.

Neutralizing antibodies have traditionally been thought to provide protection against influenza viruses. Nevertheless, the effectiveness induced by such vaccines is limited by the emergence of mutant viruses that are resistant to antibody-mediated neutralization (3). In this regard, the quest for universal influenza vaccines has fueled the interest in broadly reactive antibodies in addition to direct virus neutralizations. Antibody-dependent cell-mediated cytotoxicity (ADCC) uses effector arms of both innate and adaptive immune responses to eliminate target cells. Natural killer (NK) cells, upon triggered by specific ADCC antibodies, mediate the clearance of virus-infected cells (4). The NK cell CD16 receptor recognizes the Fc portion of IgG1 antibodies that in turn bind to antigens on virus-infected cells (5). This interaction induces degranulation of NK cells to release perforin/granzymes as well as secretion of antiviral cytokines such as interferon-γ (IFN-γ) and tumor necrosis factor-α (TNF-α) (5).

Since ADCC could invoke protective immune response against infections from a broad array of viruses, the ADCC antibody response was incorporated as one of the most important features of potential universal vaccine candidates by the World Health Organization. Notably, multiple components of influenza viruses are known to induce ADCC, including the viral surface proteins such as HA (6) and M2 ectodomain (7, 8), as well as the internal proteins including NP and M1 (9). The glycoprotein HA consists of two functional domains, the immunodominant globular head domain and the more conserved stalk domain (10). Conventionally, neutralizing antibody response to influenza virus is dominated by antibodies that target the head region, which block the virus receptor-binding site. Compared with the bulky globular HA head, the HA stem region is far less immunogenic, and antibodies directed to this domain occur at a relatively low frequency. However, a rare class of neutralizing antibodies that target a conserved site in the HA stem was reported recently, which might shed new light for the development of universal influenza vaccines (6).

We have previously identified two putative ADCC epitopes that mapped to the HA head of a pandemic H1N1 influenza virus (11). Both epitopes, designated E1 and E2, revealed by epitope mapping of convalescent-phase plasma IgG antibodies from six H1N1-infected human subjects, are dominant and highly conserved (11). In this study, we further dissected the function of the two ADCC epitopes *in vitro* and in a lethal mouse model. Surprisingly, our results demonstrated that the ADCC response elicited by the E1 epitope triggered a detrimental rather than protective effect against influenza virus infection. While the antiviral efficacy provided by the stalk-specific ADCC antibodies has been confirmed (12), our data raised concerns on the side effect of certain HA head epitopes in devising a universal influenza vaccine. In this regard, our study suggested that a delicate balance between protective immunity and over induction of ADCC should be maintained, which should be an important consideration in evaluating vaccine safety.

## Materials and Methods

### Cells and Viruses

The LA4 cell line, which was derived from mouse lung adenoma, was maintained in DMEM/F-12 medium (Gibco) supplemented with 20% heat-inactivated fetal bovine serum (FBS), 50 U/ml penicillin, and 50 μg/ml streptomycin (P/S). Peripheral blood mononuclear cells (PBMCs) were prepared by Ficoll-Paque separation (13) of heparinized whole blood obtained from healthy BALB/c mice (6–8 weeks old). To prepare the ADCC target cells, LA4 cells were transfected with an HA expression plasmid that based on the cDNA fragment of influenza virus strain A/Hong Kong/415742/2009(H1N1)pdm09. Specifically, the full-length HA fragment was cloned into a mammalian expression vector pEAK10 plasmid containing a mouse IgG1 Fc gene (CH2 + CH3) (14). The pandemic H1N1 influenza virus strain A/Hong Kong/415742/2009(H1N1)pdm09 was used for *in vitro* virus infection; while its mouse-adapted version, A/Hong Kong/415742Md/2009 (H1N1)pdm09 was propagated in embryonated hens’ eggs and utilized for *in vivo* experiment (15). The viruses were stored in −80°C in aliquot and titrated by standard plaque assay. All experiments with live viruses were conducted using biosafety level 2 facilities as described previously (16).

### Mouse Study

BALB/c female mice, 6–8 weeks old, were kept in biosafety level 2 housing and given access to standard pellet feed and water *ad libitum*. All experimental protocols followed the standard operating procedures of the biosafety level 2 animal facilities and were approved by the Animal Ethics Committee in the University of Hong Kong (17).

### Vaccination

Vaccinations were carried out to immunize the mice with E1 or E2 or HA epitopes by DNA priming and protein boost. PBS was used as a negative control. The specified vaccination scheme was listed in Table 1. To prepare the DNA plasmids, either of the E1 or E2 fragment (11) was cloned into the mammalian expression vector pEAK10 as described for the HA plasmid construction. The resultant plasmid DNA (100 μg per mice) was used for DNA priming of the mice by electroporation. To prepare protein for vaccination, recombinant HA, E1, and E2 fusion proteins were expressed in FreeStyle 293FT™ transient expression system (Invitrogen) and purified by protein A affinity (GE Healthcare). Subsequently, proteins were dialyzed and concentrated with Vivaspin 20 centrifugal concentrator (GE Healthcare), followed by protein boosting through intramuscular route. Each mouse received 25 μg protein at each protein boosting. Sera were obtained at day 68 postimmununization before virus challenge. Antibody titers raised against E1, E2, and HA in mouse sera were evaluated by ELISA as previously described with some modifications (18). Mouse sera collected from the PBS-treated group were taken as a background control.

**Table 1:** **Mouse vaccinations scheme**.

Inoculation and bleed	Day	PBS group (*n* = 15)	E1 group (*n* = 15)	E2 group (*n* = 15)	Hemagglutinin (HA) group (*n* = 15)
Bleed 0	0	6- to 8-week female mice			
Primary inoculate (DNA)	1	PBS (100 μl)	E1 plasmid (100 μg/100 μl)	E2 plasmid (100 μg/100 μl)	HA plasmid (100 μg/100 μl)
Bleed 1	28				
Boost 1 (DNA)	29	PBS (100 μl)	E1 plasmid (100 μg/100 μl)	E2 plasmid (100 μg/100 μl)	HA plasmid (100 μg/100 μl)
Boost 2 (protein)^a^	43	PBS (100 μl)	E1 protein (25 μg/100 μl)	E2 protein (25 μg/100 μl)	HA protein (25 μg/100 μl)
Boost 3 (protein)^a^	57	PBS (100 μl)	E1 protein (25 μg/100 μl)	E2 protein (25 μg/100 μl)	HA protein (25 μg/100 μl)
Bleed 2	68				
Virus challenge	69	(1,000 FPU/mouse for all groups, record the mouse body weight, and survival every day, until day 83)			

*^a^Sigma adjuvant system was added as adjuvant*.

### Virus Challenge Study

Immunized mice (15 mice/group) were inoculated with five 50% lethal dose (LD_50_) of mouse-adapted pandemic H1N1 influenza virus by intranasal route, i.e., 1,000 PFU/mouse. Animal survival and body weight were monitored daily for 14 days after virus challenge. A body weight loss of 30% was set as the humane endpoint. Three mice per group were randomly selected and euthanized on day 3 and 5 post-challenge, respectively. Mouse lungs were collected from the euthanized mice. Half of the lung tissues were harvested for virus titration by RT-qPCR methods (19), while the other half were immediately fixed in 10% of PBS buffered formaldehyde for histopathological analyses as described previously (20).

### Histopathological Assessment

Slides were examined in a blinded manner and scored with a semiquantitative system according to the relative degree of inflammation and tissue damage (21–24). Inflammation was scored as follows: 0, no inflammation; 1, perivascular cuff of inflammatory cells; 2, mild inflammation (extending throughout 25% of the lung); 3, moderate inflammation (25–50% of the lung); 4, severe inflammation involving over one half of the lung.

### ADCC Assay

Antibody-dependent cell-mediated cytotoxicity activity, reflected by the rate of cell death, was measured by a flow cytometry-based assay that described previously with some modifications (11). Generally, the PKH-67 dye (Sigma) was utilized to label the target cells, i.e., HA-transfected LA-4 cells; while 7-Aminoactinomycin D (7-AAD; Invitrogen) was used to stain the dead cells that mediated by ADCC activity. Experimentally, PKH-67-labeled target cells and unlabeled effector cells (i.e., PBMCs) were prepared in RPMI 1640 medium (Gibco) containing 10% FBS and 1% P/S with a cell density of 10^6^ and 10^8^ cells/ml, respectively. Subsequently, 50 μl of target cells were dispensed into a round-bottom 96-well plate, followed by addition of 1 μl of mouse serum. Mouse serum concentration in each group was normalized before addition according to their titers determined by ELISA. One hour after incubation, 50 μl of effector cells were added to incubate with the target cells. Three hours later, another 1 μl of 7-AAD was added to each well before performing the flow cytometry. Cell death was determined with a FACSAria III flow cytometer using the BD FACS software (BD Biosciences). Percent cell death was calculated by software analysis of four identifiable cell populations: live effector cells (no dye), dead effector cells (7-AAD positive), live target cells (PKH-67 positive), and dead target cells (PKH-67 and 7-AAD double positive). Assay controls used to define cell populations included target cells alone (background cell death) and target cells with 1 μl Triton X-100 added (maximum cell death). Percent ADCC was calculated as [(percent experimental lysis − percent spontaneous lysis)/(percent maximum lysis − percent spontaneous lysis)] × 100%, in which percent spontaneous lysis refers to the percent lysis of infected cells with effectors but in the absence of serum, while percent maximum lysis refers to the percent lysis of infected cells with effectors in the presence of 1% Triton X-100. Experiments were performed in triplicate and repeated twice for confirmation.

### Confocal Imaging

Immunostaining was performed as previously described to visualize perforin expression in mouse lung tissues (25). Rat anti-mouse perforin (abcam Ab16074) and goat anti-rat Alexa 594 were used as primary and secondary antibodies, respectively. Images were acquired with a Carl Zeiss LSM 780 system.

### Quantitative Real-time RT-PCR

RNA extraction, reverse transcription, and qPCR were performed as previously described (26, 27). In brief, total RNA was extracted from mouse lung with RNeasy Mini Kit (Qiagen) and reverse transcribed with Transcriptor First Strand cDNA Synthesis Kit (Roche). Real time PCR was performed using LightCycler^®^ 96 (Roche) machine according to the manufacturer’s instructions. Relative gene expression was normalized to the corresponding β-actin values. The sequences of the primers for RT-qPCR are listed in Table S1 in Supplementary Material.

### Statistical Analysis

Statistical comparison was performed by Student’s *t*-test using GraphPad Prism 6. Differences were considered statistically significant when *P* < 0.05.

## Results

### ADCC Responses Are Enhanced by the Sera of E1-Vaccinated Mice

**Figure 1:**
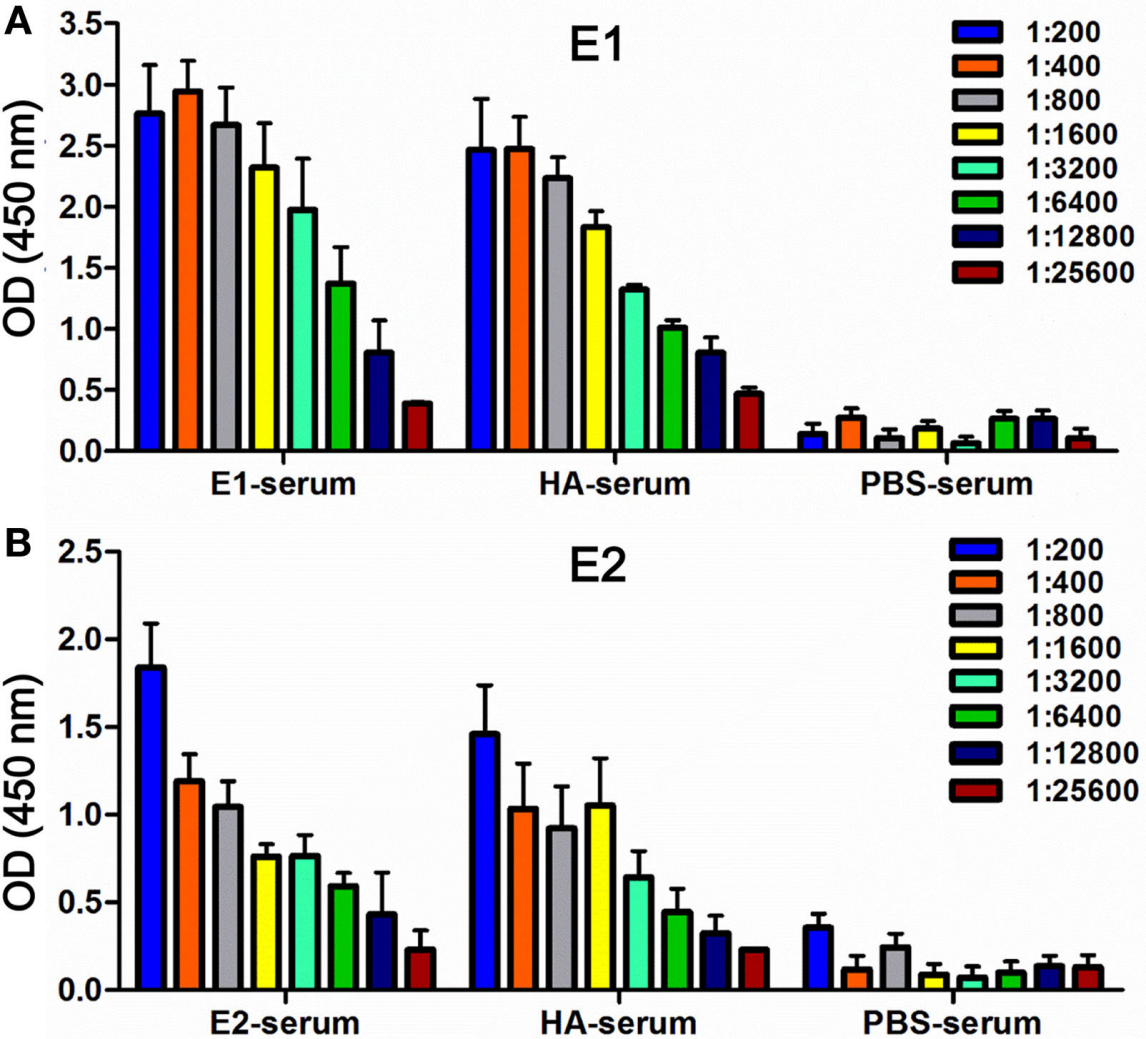
**Detection of binding activities in mouse serum samples**. ELISA was used to measure antibody titers in serum samples collected from the mice vaccinated with E1 (E1-serum), E2 (E2-serum), HA (HA-serum), or PBS (PBS-serum) on day 68. Binding was tested against either the antigen E1-Fc **(A)** or E2-Fc **(B)**, respectively. Binding intensities were measured at an absorbance of 450 nm. The experiments were conducted in triplicate. Data shown represents the mean values ± SD (*n* = 6).

In our previous study, we mapped two putative ADCC epitopes, E1 and E2, on the HA head region. By depleting E1 and/or E2 from clinical plasma IgG antibodies, the ADCC activity dropped significantly, which suggested that the two epitopes played potential roles in eliciting ADCC (11). In this study, we sought to confirm the function of these putative epitopes in the induction of ADCC activity using a mouse model. Immunization of mice gave raise to IgG titers against the E1, E2, or HA protein, as quantified by ELISA (Figure 1). A prime/boost immunization strategy was adopted, and mice that immunized with PBS or HA were included as a negative or a positive control, respectively (Table 1). Our results indicated that serum samples from mice vaccinated with E1 (Figure 1A) or E2 (Figure 1B) both exhibited strong dilution-dependent antibody responses, reaching a titer of more than 1:5000. Additionally, using HA as the coating antigen for ELISA, we demonstrated that E1 and E2 sera could bind full-length HA at a comparable efficiency (Figure S1 in Supplementary Material). Taken together, our data suggested that the vaccination was successful and the resultant serum samples could be utilized for further investigations.

**Figure 2:**
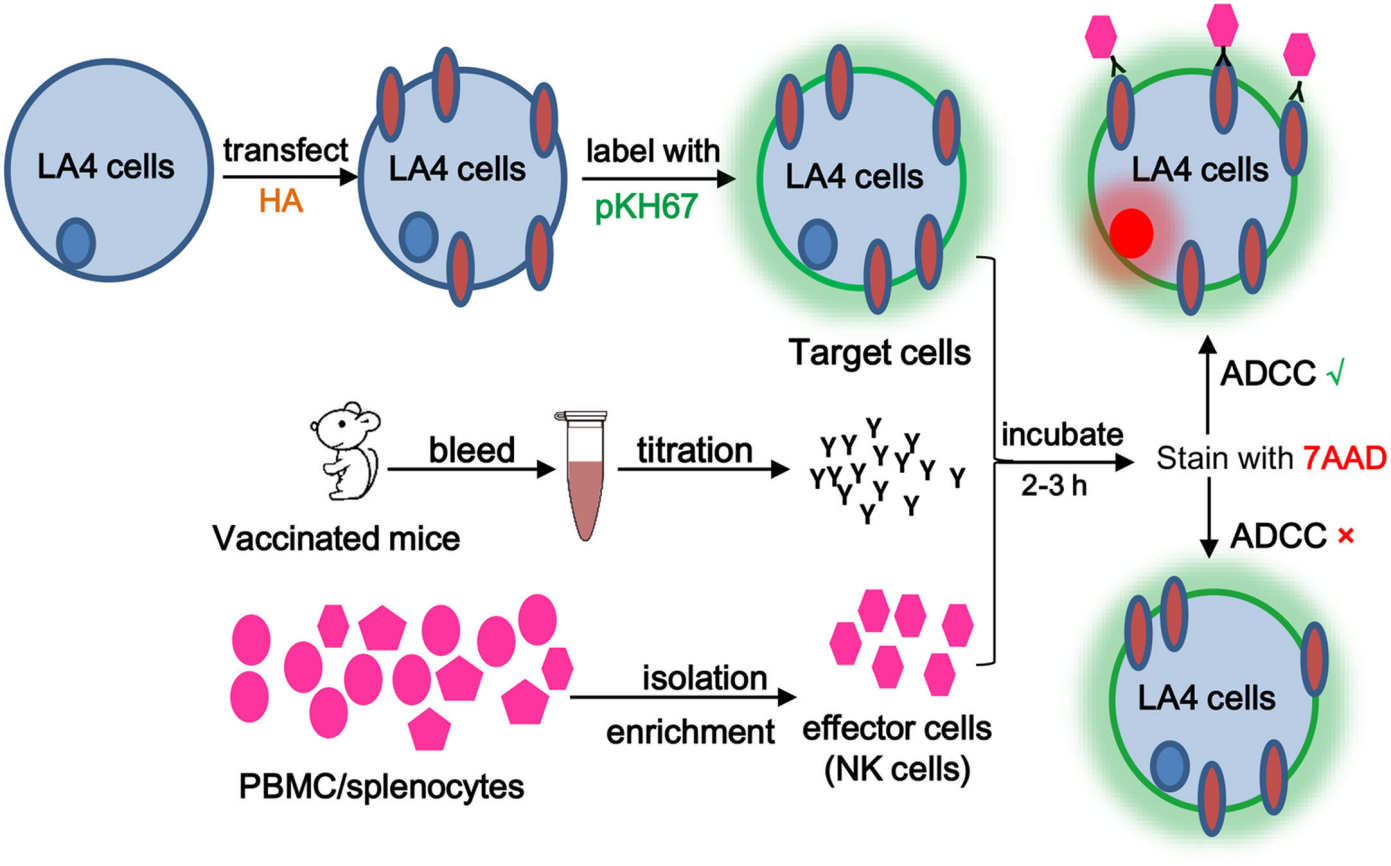
**Schematic diagram of the antibody-dependent cell-mediated cytotoxicity (ADCC) assay**. ADCC activity was determined by a flow cytometry-based assay using two fluorescent dyes. PKH-67, a membrane-labeling dye, was used to specifically identify the HA-transfected target cells. 7AAD was excluded by viable cells but could penetrate the cell membrane of dead or dying cells and intercalate into double-stranded DNA. Briefly, 50 μl of PKH-67-labeled target cells (10^6^ cells/ml) was dispensed into a round-bottom 96-well plate, followed by addition of E1/E2/HA/PBS sera and effector/PBMC cells. Following a 3-h incubation, 7-AAD was added. Cell death was determined on a FACSAria III flow cytometer using BD FACS Diva software (BD Biosciences). Percent cell death was analyzed by the Flowjo software.

**Figure 3:**
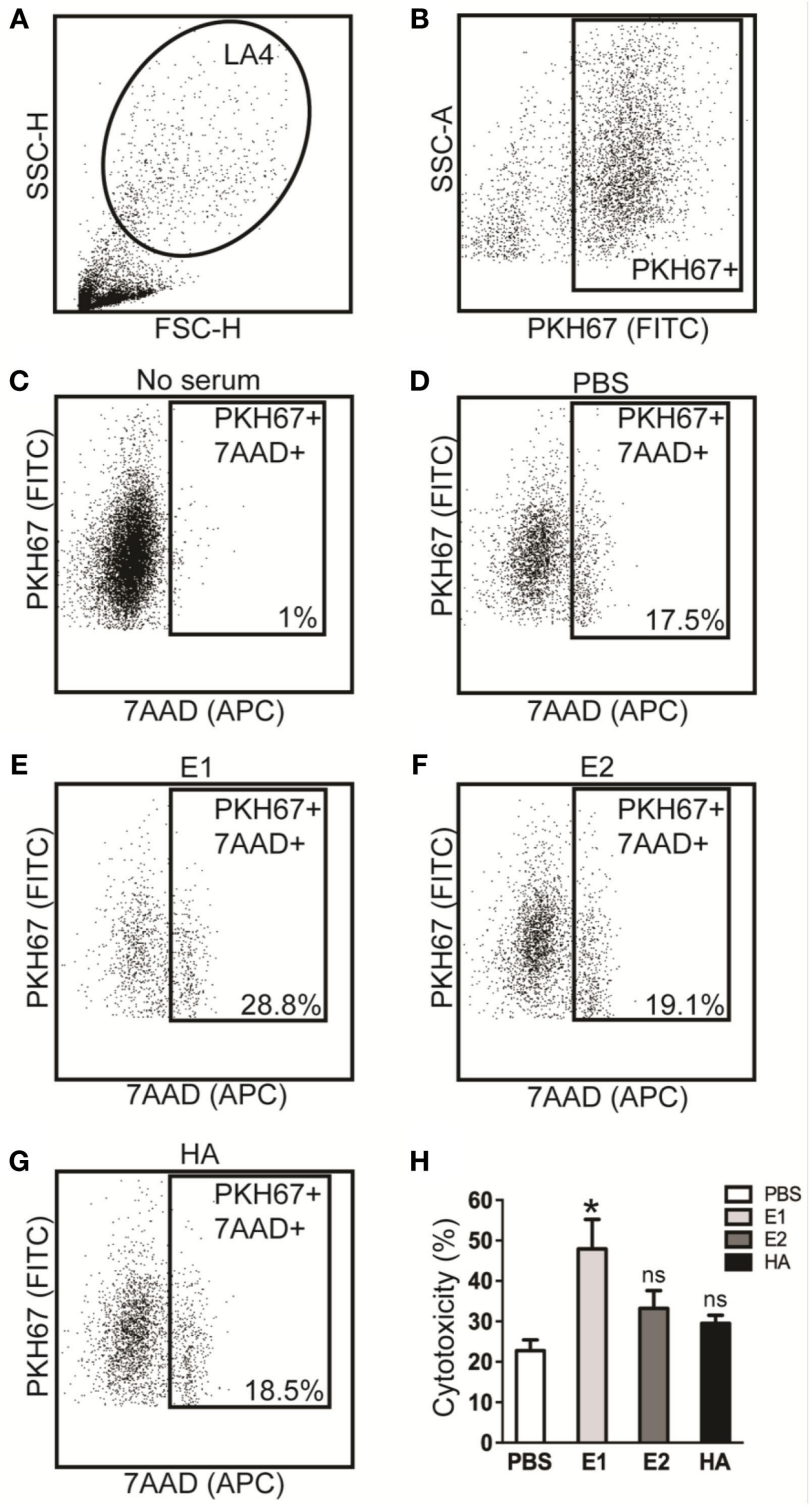
**Antibody-dependent cell-mediated cytotoxicity (ADCC) responses are enhanced by the sera of E1-vaccinated mice**. ADCC activities in serum samples collected on day 68 were tested by flow cytometry-based assays. **(A)** Gating of LA4 cells. **(B)** Gating of PKH67 positive cells. The 7AAD positive cell population for the no serum group **(C)**, PBS-vaccinated group **(D)**, E1-vaccinated group **(E)**, E2-vaccinated group **(F)**, and the HA-vaccinated group **(G)**. **(H)** ADCC activities of serum samples that collected from each group. The percent of cytotoxicity was derived based on our previously described method (11). *P < 0.05 as compared to the PBS-treated group. The results represent mean values ± SD (*n* = 5).

Next, ADCC activities in serum samples from E1-, E2-, or HA-immunized mice were evaluated by flow cytometry-based ADCC assays. To this end, the HA-transfected LA4 cells were labeled with the cell-membrane marker PKH67 and utilized as target cells for ADCC-specific antibody binding. Subsequently, the vaccinated mouse serum was added to bridge the interaction between target cells and PBMC effector cells (Figure 2). The presence of ADCC-mediating antibody was determined by analyzing the cytotoxicity rate within the cell mixture that contained the target cells, serum, and effector cells, in which the dead target cell population was revealed by the cell death marker, 7AAD (Figure 3). As shown in Figures 3A–G, sera from the E1-vaccinated mice consistently triggered the highest 7AAD + rate among all evaluated groups, suggesting that a higher percentage of cell lysis was induced in the E1 group in comparison to the other groups. The percentage of cytotoxicity was normalized using the formula reported by Srivastava et al., with spontaneous and maximum cell cytotoxicity rate taken into consideration (11). Quantitatively, sera from the E1-vaccinated mice elicited a significantly increased (*P* < 0.05) ADCC activity in comparison with the PBS-vaccinated group (Figure 3H). Of note, though sera from the E2-Vaccinated mice triggered a subtle increase in ADCC activity, the difference was not statistically significant (Figure 3H). Intriguingly, albeit HA contained the E1 epitope, sera from the HA-vaccinated group did not induce a significantly elevated cytotoxicity response in comparison to that of the PBS control group (Figure 3H).

### E1-Vaccinated Mice Are Adversely Affected by ADCC

**Figure 4:**
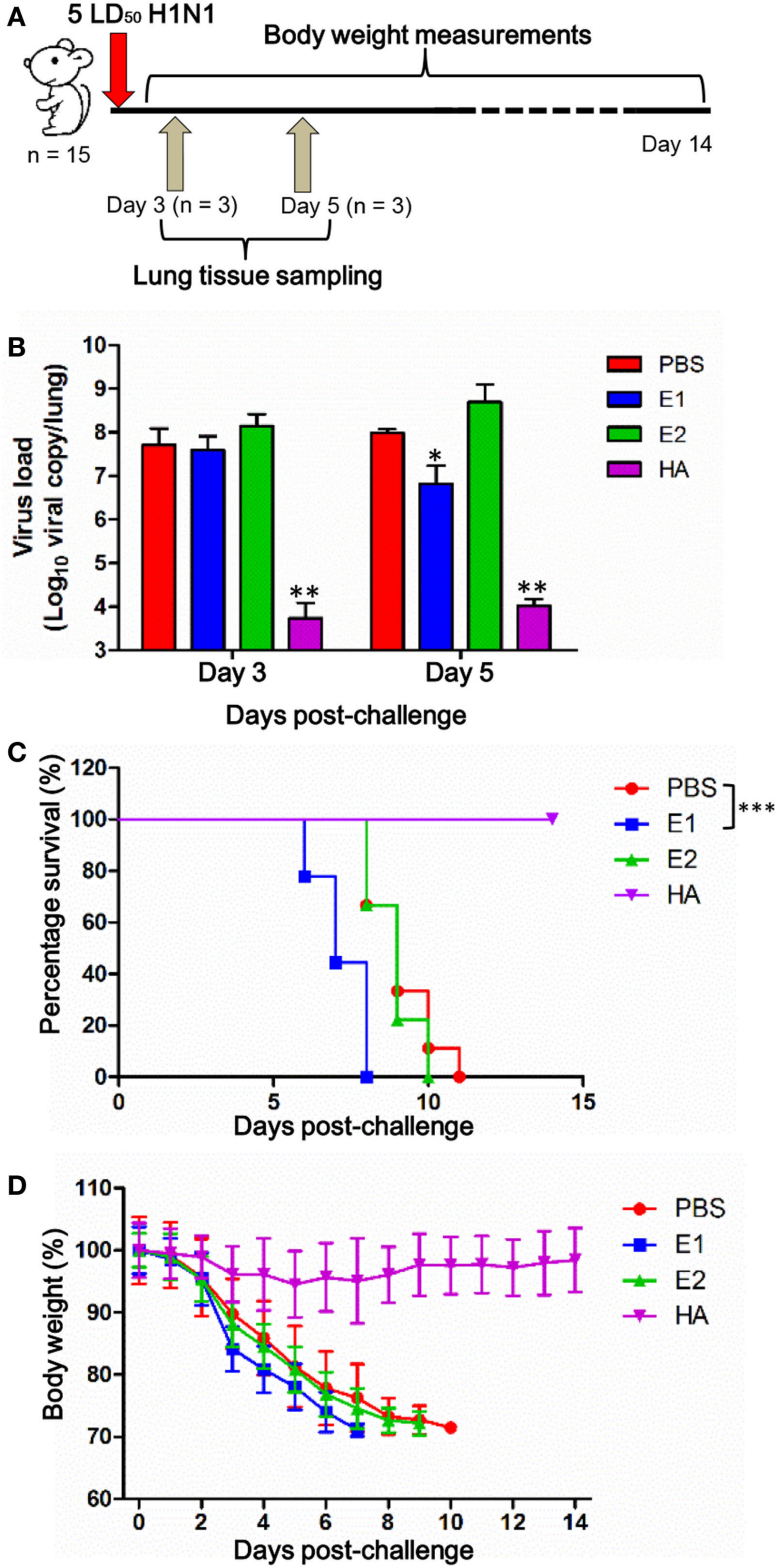
**E1-vaccinated mice are adversely affected by ADCC**. **(A)** Schematic diagram of the virus challenge study. **(B)** Three mice from each group were euthanized on day 3 and 5 post-inoculation, and lungs were collected for detection of viral loads by RT-qPCR. Results are presented as bar charts with mean values ± SD. Differences between groups were compared using the *t* test. *P < 0.05 and **P < 0.01 as compared to the PBS-immunized group. Survival rate **(C)** and body weight **(D)** of the mice (9 mice per group) vaccinated with PBS (red), E1 (blue), E2 (green), and HA (purple) were monitored for 14 days. The body weight values are shown as means ± SD for the mice that were alive at each time point (***P < 0.0001).

Since E1 was capable of inducing ADCC activities, we hypothesized that E1-vaccinated mice could potentially be protected by the elicited ADCC activity after influenza virus challenge. To this end, we inoculated the vaccinated mice with pandemic H1N1 influenza virus in a lethal mouse model (Figure 4A). As shown in Figure 4B, mice in the HA-vaccinated group, as a positive control, demonstrated a substantial reduction of viral load on both day 3 and day 5 post-inoculation in comparison to the PBS-vaccinated group. Importantly, we detected an approximately one log decrease of viral load in the mouse lungs of the E1-vaccinated group in comparison to that of the PBS-vaccinated group on day 5 post-inoculation, while no significant difference could be observed between the two groups on day 3 post-virus challenge (Figure 4B). In addition, the viral load in the lungs of the E1-vaccinated mice was notably lower on day 5 when compared with that of day 3, suggesting that the ADCC effect was triggered between day 3 and 5 post-inoculation (Figure 4B).

In parallel, we measured the survival rate and body weight changes of the mice. As shown in Figure 4C, all mice from the HA-vaccinated group survived the course of infection while all mice received PBS-treatment died, indicating that the virus challenge was successful. Unexpectedly, our results demonstrated that the mice in the E1-vaccinated group succumbed to influenza virus infection at a significantly earlier time (*P* < 0.0001) post-inoculation when compared with that of the PBS-vaccinated control group (Figure 4C). In line with the survival rate, mice from the E1-vaccinated group suffered from an apparently accelerated weight lost starting on day 3 post-inoculation in comparison to mice from the PBS- and E2-vaccinated groups, although the difference did not reach statistical significance (Figure 4D).

**Figure 5:**
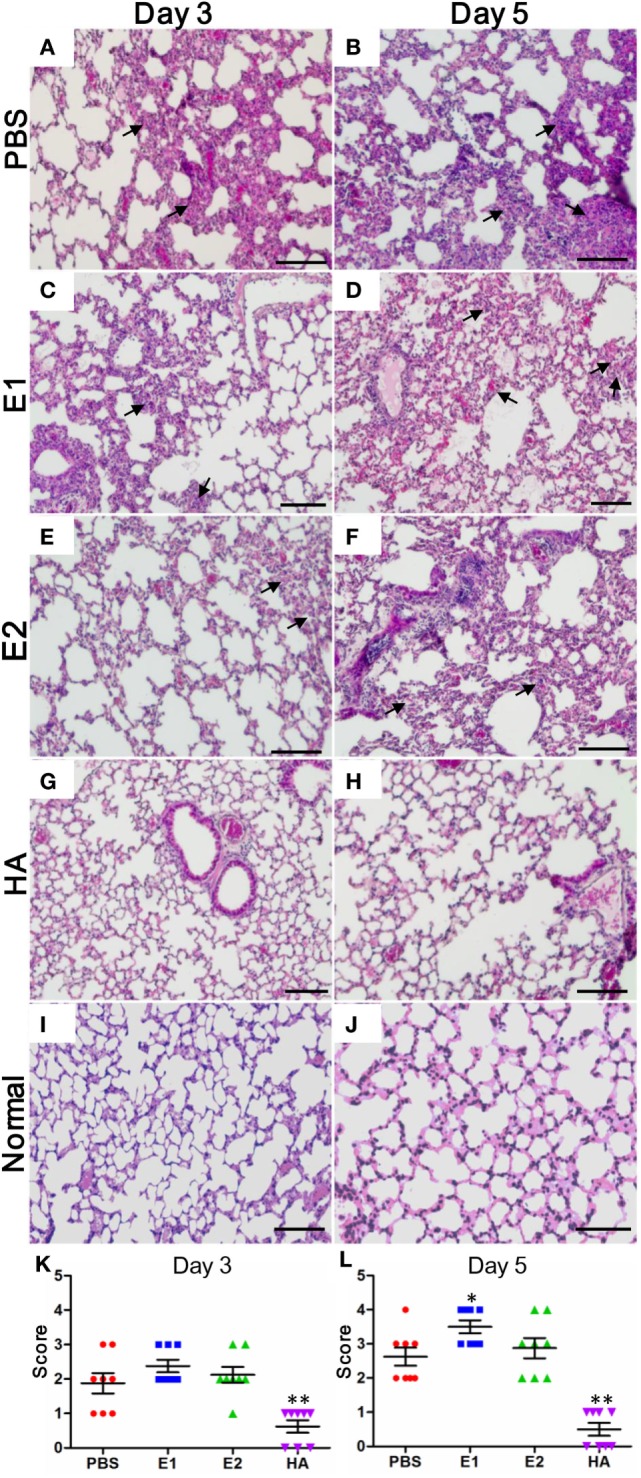
**Lungs of E1-vaccinated mice exhibit more severe histopathological changes upon influenza virus infection**. Representative histologic sections of the lung tissues from the mice harvested on day 3 and 5 post-inoculation were stained with H&E. The level of inflammatory infiltrate and thickening of the alveolar septum (as alveolar damage) was detected in samples from mice vaccinated with PBS **(A,B)**, E1 **(C,D)**, E2 **(E,F)**, and HA **(G,H)**. Lung tissues from the uninfected normal mice were included for comparisons **(I,J)**. The black arrows indicate inflammatory cell infiltration. Scale bars represent 20 μm. **(K,L)** Pathological changes were scored as the criteria indicated in Section “Materials and Methods” (*P < 0.05; **P < 0.01).

Next, we carried out histopathological examinations on the lung sections of the virus-infected mice. Using uninfected mouse lung tissues as a control (Figures 5I,J), our observation showed that on both day 3 and day 5, mice from the HA-vaccinated group (Figures 5G,H) exhibited ameliorated alveolar morphology changes when compared with those from the E1 (Figures 5C,D), E2 (Figures 5E,F), and PBS (Figures 5A,B) groups. Importantly, the lung pathological scores of mice from the HA-vaccinated group on both day 3 and day 5 were significantly lower than those of the PBS-treated mice (Figures 5K,L); while mice from the E1-vaccinated group suffered from a significantly more dramatic interstitial inflammatory infiltration than that of the PBS-treated mice on day 5 (Figure 5L). This result indicated that the detrimental lung damage of E1-vaccinated mice, possibly triggered by ADCC, might account for the reduced viral load in lungs as well as the earlier drop in survival.

**Figure 6:**
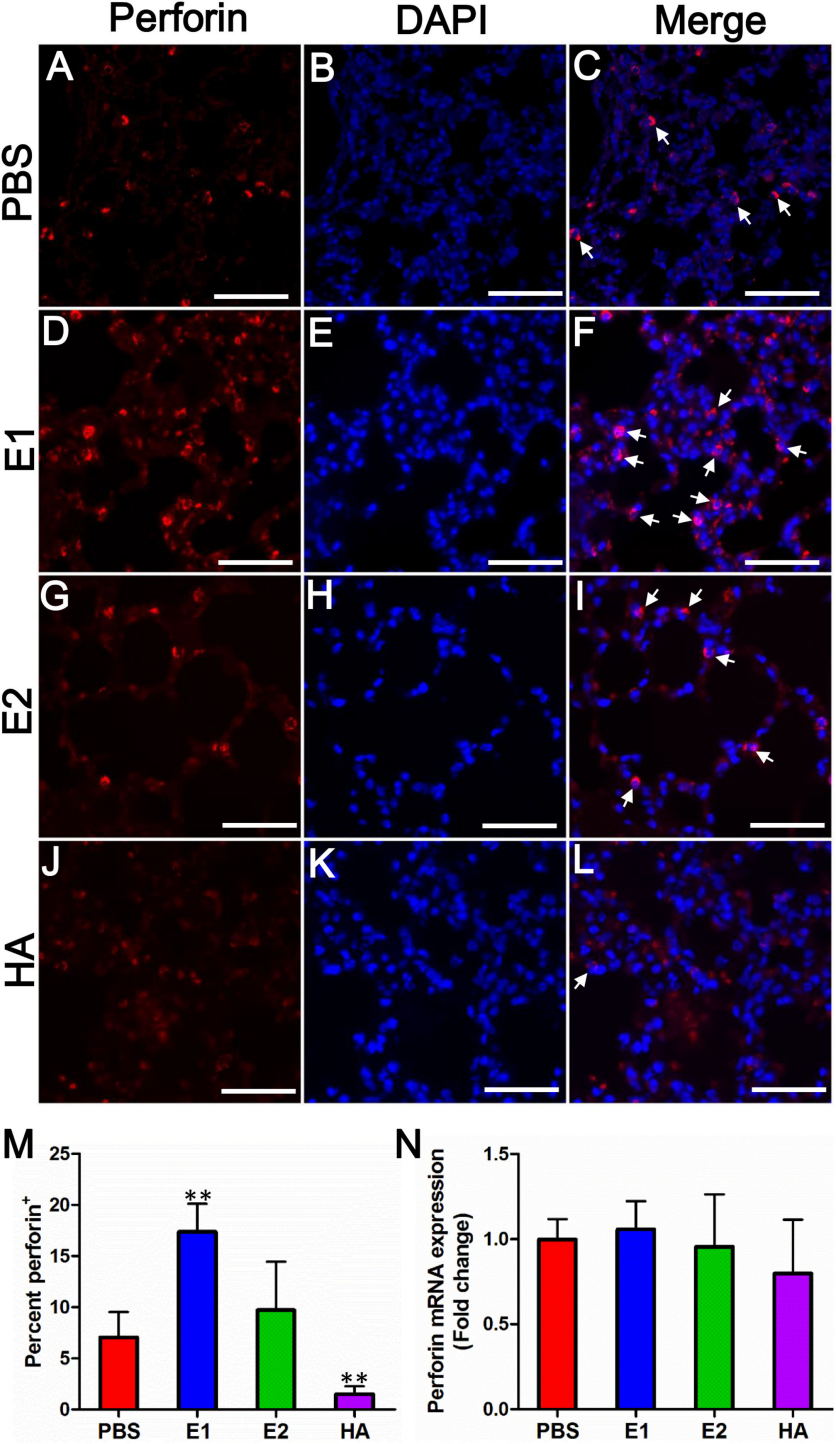
**E1-vaccinated mice express perforin at a higher level than that of PBS-, E2-, or HA-vaccinated mice upon influenza virus infection**. Perforin expression (red) from mouse lung tissues on day 5 post-inoculation was immunolabeled with rat anti-mouse perforin followed by goat anti-rat Alexa 594 **(A–L)**. Nuclei were labeled by DAPI (blue). The white arrows indicate site of perforin expressing. Scale bars represent 20 μm. **(M)** Quantification of the percentage of perforin protein expression of each group. % = (perforin positive cell/total cells) × 100 (**P < 0.01). **(N)** Quantitative real time RT-PCR comparing perforin mRNA expression levels from mouse lung tissues on day 5 post-inoculation. Data are shown as fold change compared to the perforin mRNA expression level of the PBS group. The results represent mean values ± SD (*n* = 5).

To address whether the severe lung damage in the E1-vaccinated group could be attributed to the ADCC effect, we performed immunofluorescence staining to visualize the expression level of perforin among different mouse groups. Perforin is released by activated NK cells and is known as a marker of ADCC activation (28). As quantitated in Figure 6M, the E1-vaccinated mice (Figures 6D–F) demonstrated the highest perforin expression level in the lung sections amongst the other three groups on day 5 post infection (Figures 6A–C,G–L). However, the mRNA expression level of perforin was not significantly different across all evaluated groups (Figure 6N).

**Figure 7:**
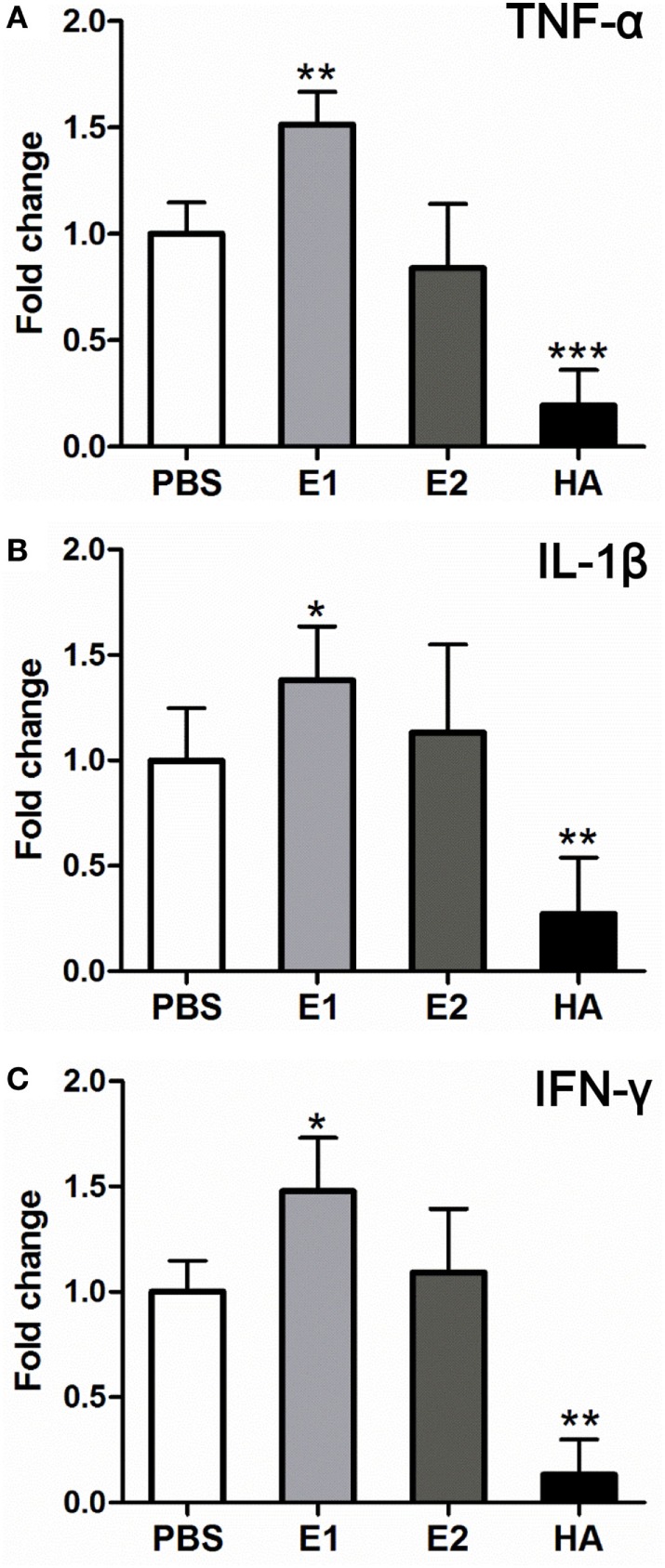
**Proinflammatory cytokines are further upregulated in the E1-vaccinated mice upon influenza virus infection in comparison with those in the PBS-vaccinated mice**. Quantitative real time RT-PCR comparing gene expression levels of TNF-α **(A)**, IL-1β **(B)**, and IFN-γ **(C)** from mouse lung tissues on day 5 post-virus challenge. Data are shown as fold change compared to the expression level of individual gene in the PBS group (*P < 0.05; **P < 0.01; ***P < 0.001). The results represent mean values ± SD (*n* = 5).

Binding of Fc receptor (FcR) on effector cells triggers the secretion of cytotoxic granules as well as antiviral cytokines and chemokines, such as IFN-γ and TNF-α (4). To investigate if elevated expression of proinflammatory cytokines might contribute to the lung damage, we measured the mRNA expression level of representative cytokines including TNF-α (Figure 7A), IL-1β (Figure 7B), and IFN-γ (Figure 7C) in the mouse lungs samples. Our results showed that the gene expression of all three proinflammatory cytokines were significantly enhanced in the E1-vaccinated group when compared with those of the PBS-treated group (Figure 7), which were in line with the perforin protein expression pattern in Figure 6. Together, our data suggested that the E1 epitope from the HA head domain mediated unfavorable ADCC that resulted in a more severe lung damage and exacerbated mouse mortality despite a successful control of the H1N1 influenza viral load.

## Discussion

Antibody-dependent cell-mediated cytotoxicity, as a bridge of the innate and adaptive immunity, plays important roles in humoral and cellular immune response (4, 9). Since ADCC antibodies are known to recognize a wide range of viral proteins that lead to the lysis of virus-infected cells, a better understanding on the ADCC mechanism during influenza virus infections will facilitate the development of universal influenza vaccines with broader protections (4, 9, 29). The conserved viral proteins or domains, such as M2 extracellular domain and HA stem domain, have been widely studied as potential targets of domain-based universal influenza vaccines. Jegerlehner and colleagues have demonstrated that the protective role of M2 ADCC-mediating antibodies was dependent on FcR (7, 8). DiLillo et al. provided further support that the influenza-specific ADCC antibody, though elicited by the HA stem, also required FcRs interaction for protection against lethal influenza infection (6). Collectively, both studies highlighted the functional importance of FcR during ADCC-mediated virus clearance. On the other side, unexpected cases have been reported that young adults without prior exposure to the 1968 H3N2 virus produced robust ADCC-mediating antibody response upon infection of this virus strain. Some individuals even possessed cross-reactive ADCC-mediating antibodies toward avian H5N1 and H7N7 strains in the absence of prior exposure (30). However, the mechanism of such antibody responses remains unclear to date.

In this study, we investigated the ADCC effect of the two putative HA head epitopes *in vitro* and *in vivo*. Our data demonstrated that E1-induced ADCC activity against H1N1 influenza virus infection *in vitro* (Figure 3). Unexpectedly, although E1 vaccination decreased the viral load in H1N1-infected mice (Figure 4B), it induced exacerbated lung damage (Figure 5) and a higher level of NK activity (Figure 6) that accelerated mouse death (Figure 4C). NK cells, which offer the first line of defense against virus infection, have been widely considered to be beneficial to the host during viral infections. However, a recent report by Zhou et al. revealed that adoptive transfer of NK cells from influenza virus-infected lungs, but not uninfected lung, resulted in a more rapid weight loss and increased mortality of virus-infected mice (31). This finding was in line with our observation that E1-induced ADCC exhibited deleterious impact to promote mortality during influenza virus infection.

Most healthy donors have a persistently low level of cross-reactive ADCC-mediating antibodies, while these cross-reactive antibodies are found in individuals in the absence of detectable neutralization (4, 9). In our previous study, both E1 and E2 epitopes were identified as putative regions that could induce ADCC activity. The depletion of such antibodies in human plasma significantly decreased the ADCC effect. However, for certain samples, it appeared that more diluted plasma exhibited higher ADCC activity than less diluted plasma, and the use of IgG antibodies at a low concentration led to a higher ADCC activity than the use of IgG antibodies at a high concentration (11). To date, there is no conclusive study on the correlation between antibody concentration and ADCC activity, neither was the optimal concentration of ADCC antibodies that could protect against virus infection elucidated. In this context, we demonstrated here that an overwhelming production of ADCC antibodies in the absence of neutralization might not play a protective role against influenza virus infection. Indeed, multiple factors such as saturation of antibodies or interference from non-ADCC antibodies may contribute to the induction of ADCC (4, 11). In this case, the threshold level of protective ADCC-mediating antibodies should be investigated in further studies.

Various ADCC assays that mainly differ in the choice of effector cells and measurement of ADCC activity have been reported (4, 9). For example, some studies used HA-transfected or virus-infected A549 cells as target cells, which were susceptible to NK cell-mediated ADCC after incubating with the sera from healthy donors or clinical blood samples (6, 32, 33). In our case, we isolated PBMCs from healthy mice as effector cells and measured the death rate of target cells in the presence of vaccinated mouse sera (Figure 2). At the same time, utilization of flow cytometry for quantitation of cell cytotoxicity provided an efficient and precise way to assess the ADCC responses (Figure 3). Importantly, the H1N1-infected LA4 cells showed a low background of cell death in the absence of antibodies (Figure 3C), which suggested LA4 as an ideal cell line for the mouse-specific ADCC assay. Collectively, the established *in vitro* ADCC assay, together with the BALC/c mouse model, might be applied for the evaluation of other influenza-specific ADCC epitopes.

Experimental mouse models are an invaluable scientific resource that allow comprehensive investigation of key biological questions *in vivo* and provide an essential platform in the study of many human diseases. It has been widely acknowledged that the mouse and human antibody repertoire share a general similarity (34–36). However, differences in germline antibody repertoire exist between species, and the number of mature naïve B cells from mice is smaller than that from humans (37). Both variations may contribute to the dissimilarities in the antibodies elicited by the E1-containing fragments in humans compared to those in mice. Due to the diversity of the B cell antigen receptor repertoire between the mouse and human model, the antibodies bind to the same fragments in distinct host models might potentially have slightly different epitopes. Alternatively, there may be fundamental differences between murine and human in terms of the regulation in NK cell cytotoxic granule secretion (38).

Surprisingly, distinct expression patterns of perforin were detected between protein (Figure 6M) and mRNA (Figure 6N) levels. The discrepancy might be explained by a previous finding that resting murine NK cells are “pre-armed” with high amounts of perforin mRNA, which can be rapidly translated into protein upon activation *in vitro* and *in vivo* (38). This mechanism of murine NK cells facilitates a better control of perforin expression, allowing a rapid production of effector proteins without the need of *de novo* gene transcription (38). Upon activation, ADCC effector cells produce various cytokines such as TNF-α, IL-1β, and IFN-γ. Further, cytokines may represent one of the arming pathways that stimulate the translation of perforin (38). Notably, human NK cell is minimally cytotoxic at rest, expresses little perforin protein, and becomes potently cytotoxic only after cytokine activation (39, 40). Our result showed that TNF-α, IL-1β, and IFN-γ were significantly elevated in E1-vaccinated group when compared with those of the PBS-treated group (Figure 7). In this regard, the upregulation of these cytokines may activate the cytotoxic perforin to cause the detrimental damage in mouse lungs.

In our previous study, E1 and E2 epitopes were located on the HA head (Figure S2 in Supplementary Material), both of which are conserved in H1N1 strains from 2009 (2009_H1N1) (11). To date, however, the role of HA head during influenza virus infection and ADCC activation has not been fully delineated. In a recent study, DiLillo and colleagues reported that neutralizing antibodies targeting the HA stem but not the HA head were capable of conferring influenza-specific ADCC (6). They proposed that only the anti-stem antibodies could bind in a correct conformation to ligate FcRs, which was based on the observation that a strain-specific anti-HA head antibody (PY102) was unable to mediate FcγR binding and to protect mice. On the other hand, HA head-induced ADCC activities were reported by a number of other groups (41–43), which was in agreement with our findings. The discrepancy between these observations might be due to the sequential and structural variations among subtypes/strains of influenza virus used. Interestingly, although both E1 and E2 epitopes are located on the HA head, serum from the HA-vaccinated group did not trigger a significantly elevated cytotoxicity response (Figure 3H), implying that additional regulators exist to control the ADCC activity.

In summary, we provided *in vitro* and *in vivo* evidence to verify the effect of the HA head epitope E1-mediated ADCC. Importantly, our data suggested that E1-mediated ADCC alone caused detrimental effect during influenza virus infection, which raised concerns on using this conserved non-neutralizing region of the HA head in future designs of a universal influenza vaccine. In fact, the “headless HA” has been recommended in several vaccine designs that aimed to make use of ADCC antibodies (44–47). Our data provided further evidence in support of this “headless HA” vaccine design strategy.

## Author Contributions

Z-WY and SY designed and performed the experiments. K-YY and HC conceived the study. LW performed the molecular modeling. ZS, MH, DY, M-YZ, and B-JZ gave conceptual advice and technical support. K-MP, CL, JZ, and HS conducted the histopathological observation and immunostaining. Z-WY, SY, HC, and K-YY wrote the manuscript.

## Conflict of Interest Statement

The authors declare that the research was conducted in the absence of any commercial or financial relationships that could be construed as a potential conflict of interest.
