# Supplementary material for: Antibody-Dependent Cell-Mediated Cytotoxicity Epitopes on the Hemagglutinin Head Region of Pandemic H1N1 Influenza Virus Play Detrimental Roles in H1N1-Infected Mice
Source: Front Immunol. 2017 Mar 21;8:317. doi: 10.3389/fimmu.2017.00317 (PMC5359280; doi:10.3389/fimmu.2017.00317)
Supplement: Supplementary file 1 [file Data_Sheet_1.docx]

**Supplementary Materials**

**Antibody-Dependent Cell-Mediated Cytotoxicity Epitopes on the Hemagglutinin Head-region of Pandemic H1N1 Influenza Virus Play Detrimental Roles in H1N1-Infected Mice**

**Authors:** Zi-Wei Ye^1*^, Shuofeng Yuan^1*^, Vincent Kwok-Man Poon^1^, Lei Wen^1^, Dong Yang^1^, Zehua Sun^1^, Cun Li^1^, Meng Hu^1^, Huiping Shuai^1^, Jie Zhou^1,2^, Mei-Yun Zhang^1^, Bojian Zheng^1^, Hin Chu^1,2#^, Kwok-Yung Yuen^1,2,3,4#^

**There are 2 supplementary figures and 1 supplementary table.**

**Figure S1** Detection of binding activities in mouse serum samples using full-length HA-Fc protein as antigen.

**Figure S2** The localization of E1 and E2 on the HA protein.

**Table S1** Oligonucleotides for RT-qPCR in this study.

**Supplementary Figure legend**





**Figure S1: Detection of binding activities in mouse serum samples using full-length HA-Fc protein as antigen.**

ELISA was used to measure antibody titres in serum samples collected from the mice vaccinated with E1 (E1-serum), E2 (E2-serum), HA (HA-serum), or PBS (PBS-serum) on day 68. Binding was tested against the antigen HA-Fc. Binding intensities were measured at an absorbance of 450 nm. The experiments were conducted in triplicate. Data shown represents the mean values ±SD (n = 6).


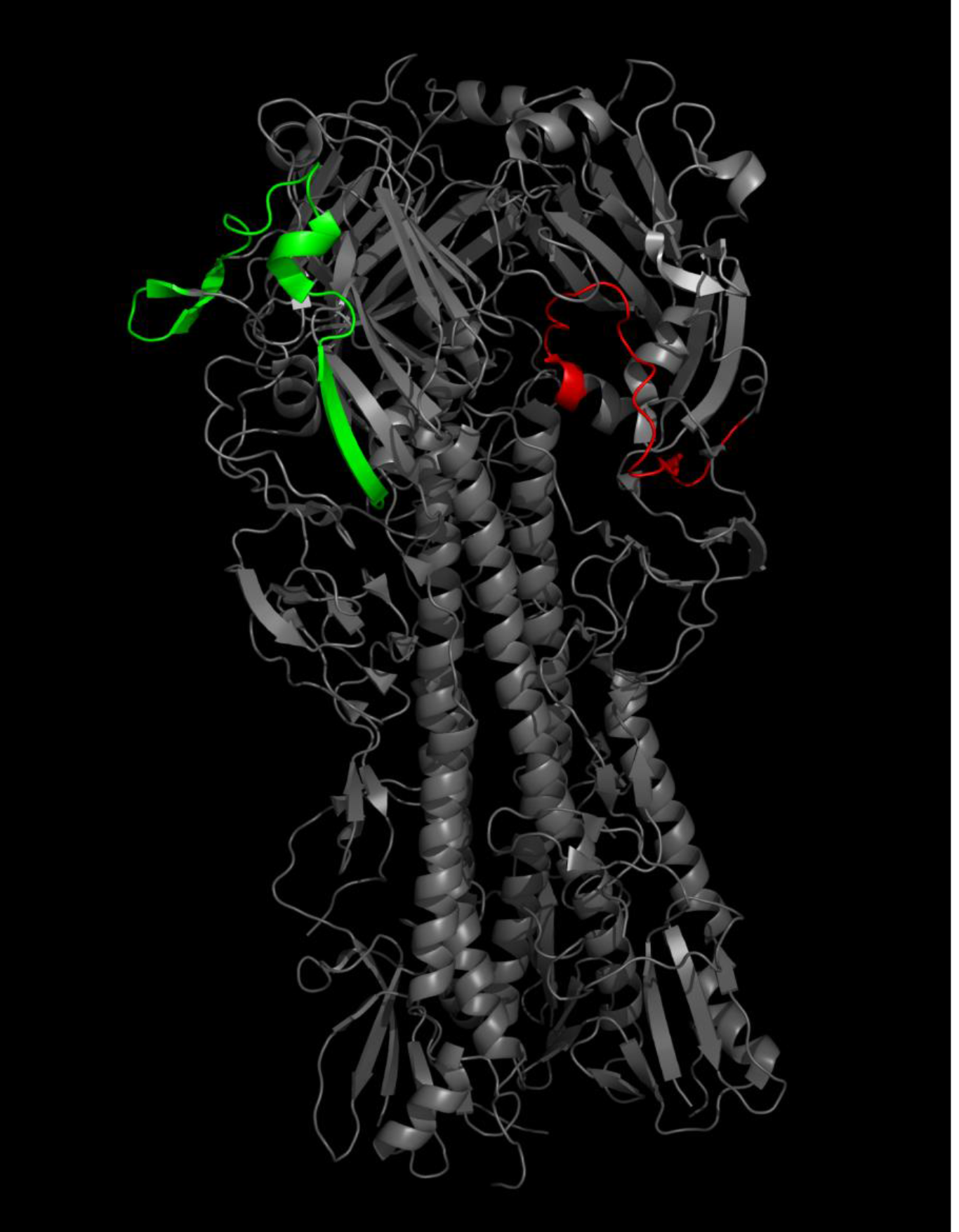


**Figure S2: The localization of E1 and E2 on the HA protein.**

Schematic representation of the epitopes that induce ADCC effect on the globular head of 2009 pandemic H1N1 HA protein. Epitopes E1 and E2 are highlighted in red and green color respectively. Images were created with PyMol 1.84, and the HA structure was obtained from the Protein Data Bank (PDB accession number 4JU0).

**Table S1 Oligonucleotides for RT-qPCR in this study.**

| Gene name | Orientation | Sequence (5’ – 3’) |
| --- | --- | --- |
| β-actin | Forward | ACGGCCAGGTCATCACTATTG |
| β-actin | Reverse | CAAGAAGGAAGGCTGGAAAAG |
| Perforin | Forward | CGTGAGCGTCACGTCGAA |
| Perforin | Reverse | GTTCCCGAAGAGCAGATCATG |
| TNF-α | Forward | ATAGCTCCCAGAAAAGCAAGC |
| TNF-α | Reverse | CACCCCGAAGTTCAGTAGACA |
| IL-1β | Forward | GCCTTGGGCCTCAAAGGAAAGAATC |
| IL-1β | Reverse | GGAAGACACAGATTCCATGGTGAAG |
| IFN-γ | Forward | AAGCGTCATTGAATCACACC |
| IFN-γ | Reverse | CGAATCAGCAGCGACTCCTT |
